# Response to: Commentary on: The Best Under Stress: An Analysis of Breast Tissue Expander Response to External Forces

**DOI:** 10.1093/asjof/ojad044

**Published:** 2023-05-22

**Authors:** Daniel Najafali, Farrah C Liu, Karanvir Raman, Bhagvat Maheta, Golddy Milagros Saldana, Lucas Heldman, Priscila Cevallos, Rahim Nazerali

We appreciate the reader's input and thoughtful commentary on our recent article on the deformational forces of 3 commercially available tissue expanders (TEs).^1,2^ The author raises a valid point that advancements in reconstructive plane utilized, namely prepectoral, alter the intracorporal forces on a TE. This is an excellent observation of changes in practice and procedural technique which has influenced the impact of the pectoralis major muscle on the breast soft-tissue pocket. We agree that surgeons should bear in mind this practice development while performing breast reconstruction. However, expander selection is multifactorial and an essential part of the reconstructive decision-making process. Understanding the response of a TE under both extra-corporal and intracorporal forces is essential for making informed and patient-specific selections. In a 2019 review article titled, “The bioengineered prosthetic breast reconstruction: advancements, evidence, and outcomes,” the reviewer did note that “proper device selection is critical and optimized with biodimensional planning such that the device will closely match the footprint of the breast or mastectomy pocket.”^3^ An understanding of implant selection after expansion is necessary to ensure the appropriate pocket dimensions match the footprint of the breast implant within the post-mastectomy soft-tissue envelope.

Patient selection and a surgeon's comfort with their technique and equipment are among the most critical factors when weighing reconstructive modalities. However, it is important to understand how daily stresses applied to the TE and chest alter the final breast pocket. Having this understanding allows the surgeon to best select an implant that mimics the final dimensions of the postexpansion pocket. This analysis provides additional information for understanding the impacts of extra-corporal forces on the TE. Daily activities do contribute to changes in force on the expander and resultantly the chest wall, soft tissue, and capsule created when the expander deforms under pressure. We must take these changes into account when selecting a future prosthesis to match the breast pocket. Our study's results provide additional information about the attributes of commercially available TEs to allow surgeons to select the most appropriate device based on their desired characteristics. This decision, based on the forces and deformational responses outlined in our study, can be based on the desire to have a more structured TE compared with one that provides increased displacement. Some surgeons dislike this property, but it can be of imperative necessity when developing a stable pocket for a final implant. Anterior–posterior stress applied to the pocket translates to pocket deformation in the horizontal plane. The selection of different TEs modulates these forces and can allow for a better approximation of the final implant width (and height when selecting a round silicone or saline implant). Irrespective of the initial expander selected, it is important to understand the natural movement of the capsule and pocket during activities such as hugging, sleeping, wearing garments such as compressive breast binders or bras, and months of force applied through daily activities. Additionally, pressure (force/unit area) is a key mediator of mastectomy flap necrosis, and its effects can be altered by changing the fill pattern of the TE. Studies in this arena may pave the way for future studies on the effect of mastectomy skin flap on TE behavior.

Our study aimed to give surgeons increased precision when selecting a TE and parameters to assist in predicting the behavior of the prosthesis in relation to the final breast pocket. Despite the decreased intracorporal forces that a TE is subjected to with prepectoral placement, there are still extra-corporal pressures placed on the prosthesis that can impact malposition or patient outcomes. These forces should not be viewed as negligible, as they can impact the breast pocket's footprint and should be considered during final implant selection. Base diameter is arguably the most important part of the selection criteria for a TE as well as a final implant and lateral displacement is important to consider to ensure base width is appropriately accounted for. Selecting an implant that matches the base width decreases the movement of the implant in the pocket and provides a tighter fit.
